# Evaluation of the expression pattern of TIAs pathway genes in response to tryptophan amino acid treatment and drought stress in *Catharanthus roseus*

**DOI:** 10.1371/journal.pone.0333313

**Published:** 2025-10-09

**Authors:** Farshid Yousefi, Alireza Abdali Mashhadi, Amin Lotfi Jalal Abadi, Alireza Shafeinia, Saied Jalily, Narges Soltani

**Affiliations:** 1 Production Engineering and Plant Genetics Department, Faculty of Agriculture, Agricultural Sciences and Natural Resources, University of Khuzestan, Mollasani, Khuzestan, Iran; 2 Department of Water Engineering, Agricultural Sciences and Natural Resources, University of Khuzestan, Mollasani, Khuzestan, Iran; 3 Production Engineering and Plant Genetics Department, Faculty of Agriculture, Agricultural Sciences and Natural Resources, Lorestan University, Khorramabad, Lorestan, Iran; UNC CH: The University of North Carolina at Chapel Hill, UNITED STATES OF AMERICA

## Abstract

The leaves of the medicinal-ornamental plant *Catharanthus roseus* serve as the exclusive source of the anticancer alkaloids vinblastine and vincristine. The limited synthesis of these alkaloids, alongside efforts to enhance their production, has consistently been a focal point of research. Water scarcity, recognized as one of the most significant constraints in agriculture, has prompted this study to examine the effects of the amino acid elicitor tryptophan and drought stress on the alterations in secondary metabolites of *C. roseus* and the genes implicated in their biosynthetic pathways. This investigation was factorial experiment conducted within a completely randomized design (CRD) with three replications. The first factor involved drought stress (40% and 100% field capacity), the second factor pertained to tryptophan concentrations (0 and 250 ppm), and the third factor encompassed duration (24, 48, 72, and 168 hours). Key genes associated with four metabolic pathways, phenolic/flavonoid, indole, terpenoid, and alkaloid pathways, were analyzed using quantitative polymerase chain reaction (qRT- PCR). Notably, the *Cm* gene (phenolic/flavonoid pathway) exhibited increased expression across all treatments, with the highest expression level recorded at 168 hours under the combined conditions of tryptophan and drought. Genes associated with the indole alkaloid pathway (*As* and *Tdc*) demonstrated similar temporal variations, with peak expression levels observed at 24 hours, particularly under drought stress. Genes within the terpenoid pathway (*Sls*) and alkaloid pathway (*Str*, *Dat*, *Prx*) displayed an initial increase in expression at 24 hours, followed by a decline at 48 and 72 hours, and a subsequent increase at 168 hours post-treatment in comparison to the control. Additionally, alkaloid accumulation (vincristine, vinblastine) significantly increased, especially under severe drought stress, correlating with the observed gene expression patterns. Non-enzymatic antioxidants, including phenols and flavonoids, also exhibited elevated levels in response to stress and tryptophan treatment. Furthermore, tryptophan application resulted in a doubling of plant biomass compared to the control. Collectively, the findings of this study suggest that the combination of drought stress and tryptophan application modulates gene expression and metabolite production in *C. roseus*, which may be crucial for optimizing alkaloid biosynthesis under drought stress conditions.

## Introduction

**Fig 1:**
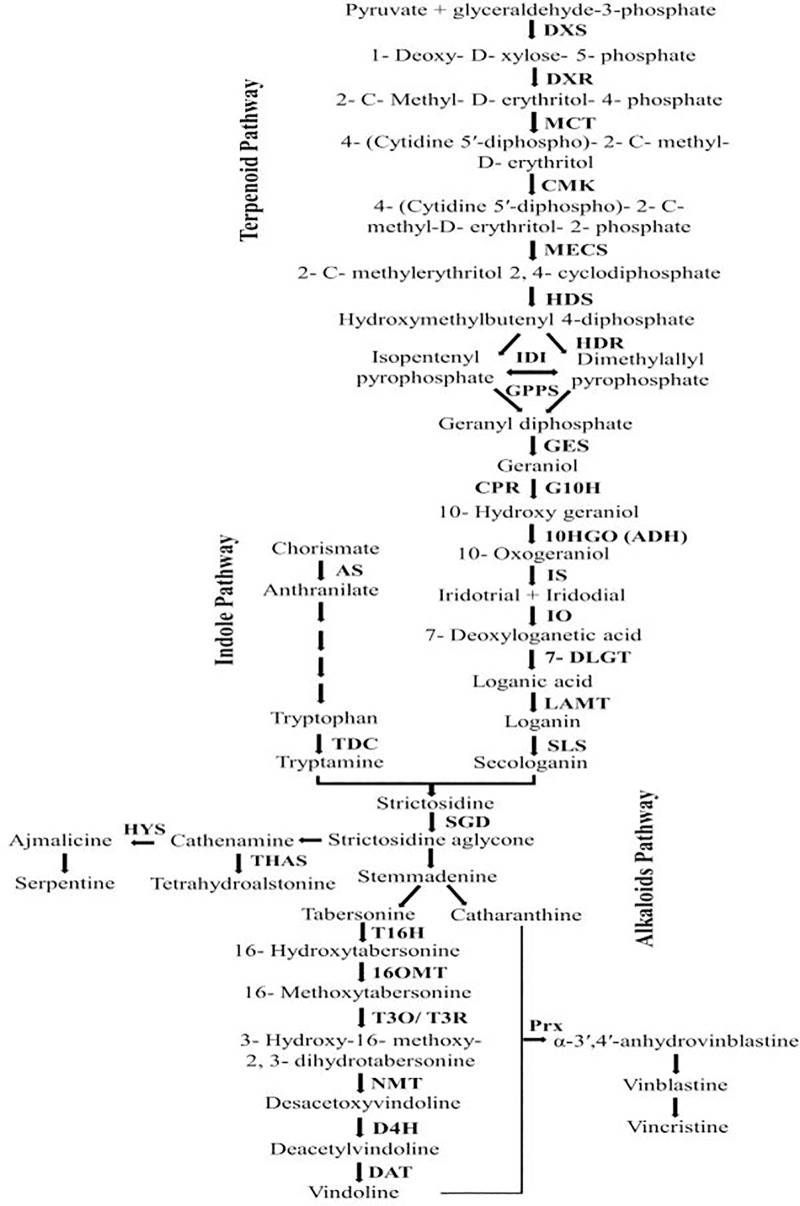
Synthesis pathway of terpenoid indole alkaloids in leaf tissue [17]. TIAs pathway consists of three indole pathways, terpenoid pathway and alkaloid pathway, the final products of terpenoid and indole pathways enter the alkaloid pathway with the activity of STR enzyme and make compounds of indole terpenoid alkaloids in leaf and root tissues.

While many drugs can be chemically synthesized, vinblastine sulfate and vincristine sulfate are exclusively derived from the leaves of the Periwinkle plant. The Periwinkle (*Catharanthus roseus* L.), is an ornamental and medicinal plant from the Apocynaceae family, indigenous to tropical and subtropical areas like South India, Indonesia, and Madagascar [1–4]. These anticancer alkaloids are synthesized in the terpenoid indole alkaloid pathway (TIAs). The TIAs include the indole, terpenoid, and alkaloid pathways. Tryptamine, originating from the tryptophan precursor in the indole pathway, along with secologanin from the terpenoid pathway, are combined by the strictosidine synthase enzyme to enter the alkaloids pathway, resulting in the creation of strictosidine (Fig 1). This compound is then converted into vindoline, vinblastine, and vincristine through the action of downstream genes and enzymes within the plant’s leaf tissue [4–6]. The TIAs pathway’s functionality is affected by upstream gene alterations and their product levels. Thus, employing an elicitor like the amino acid tryptophan, which is a tryptamine precursor, can modify the expression patterns of downstream genes within this pathway. Tryptophan, an aromatic amino acid, is transformed into tryptamine by the tryptophan decarboxylase (*TDC*) enzyme, serving as a precursor for various alkaloids, including melatonin, sumatriptan, eletriptan, harmine, vinblastine, vincristine, ajmalicine, and serpentine [7]. Plant metabolite production, particularly alkaloids, is significantly influenced by growth stages and environmental conditions [5,8]. Abiotic stresses, such as drought, can severely impact plant performance, leading to reduced photosynthesis, smaller leaf surface area, deeper root systems, and the generation of antioxidant enzymes to counteract reactive oxygen species [9]. The alkaloids production pathway in the Periwinkle plant are sensitive to abiotic stresses like drought, temperature, salinity, air pollution, heavy metals, pesticides, and soil pH. However, certain elicitors can mitigate stress effects [9]. Some amino acids, including tryptophan, can conserve plant energy, alleviate drought stress, and boost medicinal metabolite production [5,10–12]. Beyond being a secondary metabolite precursor like alkaloids, tryptophan also the precursor of auxins, external application of tryptophan has shown to be more beneficial for plant growth and performance than auxin. As an osmolyte and ion transport regulator, tryptophan contributes to stomatal regulation and cell detoxification. Foliar application of tryptophan during drought stress not only serves as an osmolyte but also enhances auxin production, promoting root growth and ultimately aiding in drought stress mitigation and enhancing plant stress tolerance [11,13,14]. Experiments with foliar application of tryptophan and salicylic acid on corn plants have demonstrated that these treatments can lessen drought stress effects, improving relative water content, leaf membrane stability index, chlorophyll, and potassium levels. Notably, plants treated with 100 ppm salicylic acid and 15 ppm tryptophan exhibited significantly better results compared to other treatments and control plants [11]. In the indole pathway of TIAs, both tryptophan decarboxylase and anthranilate synthase play crucial roles. Research on the hairy roots of the Periwinkle plant has shown that overexpressing the anthranilate synthase gene, under the control of an inducible promoter, leads to increased transcription of this gene. This, in turn, enhances the transcription of the downstream *Tdc* gene, reaching its peak 12 hours post-induction of anthranilate synthase. Subsequently, the expression levels of the *Tdc* gene begin to decline, albeit at a very gradual and nearly constant rate [4]. Further studies have revealed that the transcription of the *Tdc* gene in Periwinkle seedlings is augmented by methyl jasmonate (MeJA), with the transcription levels showing a progressive increase. The highest transcription levels were observed 48 hours after treatment with 0.2 and 2 mM concentrations of MeJA [15]. When examining Periwinkle cell cultures, treatments with MeJA, cyclodextrin, and their combination demonstrated effectiveness in stimulating *Tdc* gene expression. Notably, the combined treatment of MeJA and cyclodextrin had a more pronounced effect on enhancing *Tdc* gene transcription than either treatment alone. These treatments also upregulated the transcription of genes in the TIAs terpenoid pathway (*G10h* and *Sls*) and increased the production of the alkaloids ajmalicine and catharanthine [16]. Given the intricate and multistep nature of their synthesis and the fact that the Periwinkle plant is the sole source of the vital alkaloids vincristine and vinblastine, understanding the genetic pathways for the production of indole terpenoid alkaloids is of paramount importance [1–3]. The TIAs pathway, comprising indole, terpenoid, and alkaloid routes, is subject to the influence of upstream gene expression changes, which ultimately affect downstream gene expression patterns. Therefore, analyzing the expression patterns of the initial and terminal genes of these three pathways can provide deeper insights into the synthesis process. The current study investigated the gene expression patterns of all three pathways indole (*Sls*), terpenoid (*As* and *Tdc*), and alkaloids (*Str*, *Dat*, and *Prx*) as well as the flavonoid and Phenylpropanoid (*Cm*) synthesis pathways in the Periwinkle plant under the treatment of tryptophan amino acid and drought stress.

**Fig 2:**
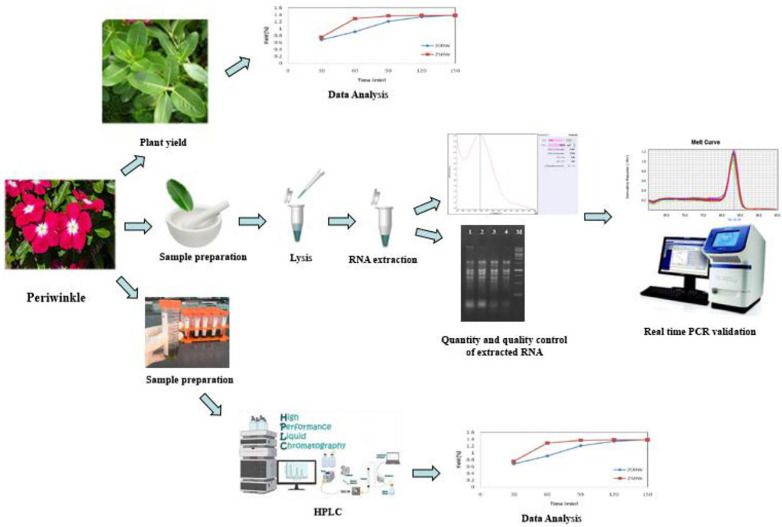
Schematic view of the poplin related to the study stages. In three parts: physiological (Total Yield), Gene Expression, and biochemical (Non-Enzymatic Antioxidants: phenol, Total Flavonoid Content, vinblastine and vincristine alkaloids).

## Material and treatments

**Fig 3:**
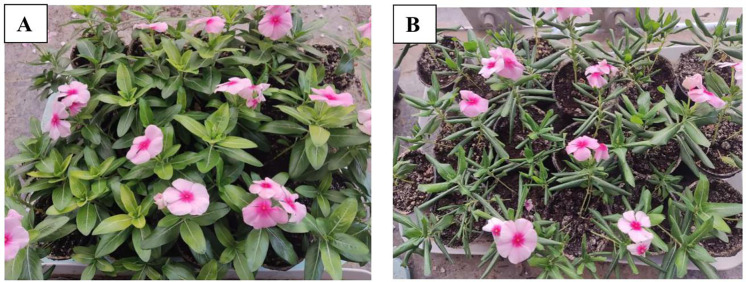
Seedlings of *C. roseus.* A: Control seedlings and B: Seedlings under drought stress of 40%.

The pipeline of study is shown in Fig 2.  Periwinkle seedlings were disinfected with hypochlorite for (1 min) in culture trays. After reaching the four-leaf stage, they were planted in pots with dimensions of 30 cm in height and 15 cm in diameter, containing a mixture of field soil, rotten manure, and sand (in a 1:1:1 ratio). The treatment was carried out from the eight-leaf stage to the beginning of the flowering phase. This study was conducted as a factorial experiment in a completely randomized design with three factors: foliar application of the amino acid tryptophan at two levels (0 and 250 ppm), drought stress (at two levels: 40% and 100% of field capacity), and time (at four intervals: 24, 48, 72, and 168 hours) (Fig 3). Foliar spraying of tryptophan amino acid was done at the eight-leaf stage and repeated 30 days later. Two days after the first foliar application, drought stress was applied and continued for one month until flowering. Leaf sampling occurred at 24, 48, 72, and 168 hours after the second foliar spraying, with three biological replicates for each sampling point.

### Gene expression

#### RNA extraction and cDNA synthesis.

Total RNA was extracted from 50 mg of powdered leaf tissue from each sample using the DENA Zist AsiA Column RNA isolation Kit (Cat. No. S-1021–1). The quantity and quality of the extracted RNAs were assessed using a NanoDrop 2000c Spectrophotometer (Thermo Scientific NanoDrop 2000, USA) and 1% agarose gel electrophoresis. Prior to cDNA synthesis, DNase I treatment was performed following the protocol of SinaClon (Cat. No. MO5401) in a final volume of 10 μl. Subsequently, the first strand of cDNA was synthesized using the Anacell kit (Lot n: CS0025) and an oligo-dT primer from 500 ng of RNA, in a final volume of 20 μl.

#### Real-time PCR analysis.

Real-time PCR was conducted using the Step One Plus Real-time PCR system (Applied Biosystems, USA) and SYBR Green High ROX (Ampliqon Cat. No. A325402). Specific primers for each gene as listed in (Table 1) by [4,17,18] were used in three biological replicates and three technical replicates.

**Table 1:** Name, sequence, annealing temperature, accession number and amplified fragment of primers.

Gene Name	Primer Sequence (5′-3′)	Ta (°C)	Accession	PCR Product (bp)
*Cm*	F: GGGTGATGCCTTTAACCAAA	60	FD419102.1	159
	R: GCGACATTGTATCGTGAAGC			
*As*	F: TGAGAAGGTGGAGAAACTGATG	60	AJ250008.1	110
	R: GTAGAACGTCCCAGCTAGTTAAG			
*Tdc*	F: CCTGTATATGTCCCGAGTTTAG	60	X67662.1	153
	R: TCGTAGTGAGTGCCCTTAG			
*Sls*	F: CACGAGGACACAAAGTTAGG	60	KF415117.1	161
	R: TTGTTCTTGGTGGCATTGG			
*Str*	F: CGCCTACGCATCTCCCTTCT	60	X53602.1	82
	R: TGTCCTCCCACACAATGGTCT			
*Dat*	F: AGCACAAATGGAAGAGTTCG	60	LN809930.1	158
	R: AGGGTTGAAACAAGCATACC			
*Prx*	F: GCAACATCTCCCAGACCACA	60	KT032115.1	117
	R: GTTCTCCCAACACTATGAGCACC			
*Rsp9 (Ref. gene)*	F: GAGGGCCAAAACAAACTTGA	60	AJ749993.1	145
	R: CCCTTATGTGCCTTTGCCTA			

In each reaction, we combined the following components:

- 40 ng/μL cDNA (8 μL from a starting concentration of 100 ng/μL)
- 0.4 μM of both forward and reverse primers
- SYBR Green PCR Mastermix (1X)

The final reaction volume was 20 μL. The thermal cycling conditions included an initial denaturation step at 95°C for 10 minutes, followed by 40 cycles of denaturation at 95°C for 30 seconds, annealing at 60°C for 20 seconds, and extension at 72°C for 20 seconds.

We determined the relative expression levels using the threshold cycle (CT) data and applied the ΔΔCT method with REST^®^ software.

### Evaluation of vinblastine and vincristine alkaloids

#### Sample preparation and alkaloid extraction.

We collected leaf samples from both the 3-day and 7-day treatments to quantify the levels of vinblastine and vincristine alkaloids. To prepare the samples, we utilized a methanol: water (80:20) mixture as the extraction solvent, following established procedures [19,20]. The leaf samples were air-dried in the shade. Next, we added 1 ml of n-hexane to 0.5 g of powdered leaf tissue. After evaporation of n-hexane, extracting solvent was added to the samples in three stages of 24 hours and the extracts of all three stages were combined together. After evaporation of the methanol in the extraction solvent, 10 ml of distilled water was added to the sample and the pH was adjusted to 3.5 using hydrochloric acid, conducting three rounds of washing with chloroform and collecting the aqueous phase containing the alkaloids each time. The pH of the collected aqueous phase was then raised to 8.5. We added chloroform and stirred the sample, collecting the chloroform phase (three times). Finally, we dried the collected chloroform phase and dissolved the dried samples in methanol for subsequent High-Performance Liquid Chromatography (HPLC) analysis.

#### Quantification of vinblastine and vincristine alkaloids by HPLC.

High-performance liquid chromatography (HPLC) was performed using Eurospher II 100–5 C18 column with precolumn (Column 250 * 4.6 mm) and UV detector (model K-2600) with HPLC device (KNAUER WellChrom model) at 254 ɳm. The mobile phase consisted of Acetonitrile: Sodium dihydrogen phosphate (0.1 M) containing 0.5% acetic acid with a composition of 21:79 (pH = 3.5) and a flow rate of 1 mL/min [21]. Subsequently, we analyzed the data using SAS software (version 9.4) and performed analysis of variance and mean comparison (by the least significant difference method (Duncan’s)) for this section.

### Evaluation of non-enzymatic antioxidants

#### Measurement of total phenolic content.

The total phenolic content was measured using the Folin-Ciocalteu colorimetric method with gallic acid as the standard. The extract was obtained by extracting 1 gram of dry leaf weight in 96% pure ethanol. To 0.1 mL^-1^ of the plant extract or standard solutions (concentrations of 0–100 mg mL^-1^ gallic acid in distilled water), 2.8 mL^-1^ of distilled water and 0.1 mL^-1^ of diluted Folin-Ciocalteu reagent (1:10 v/v) were added. After 5 minutes, 2 mL^-1^ of 7.5% sodium carbonate solution were added to the mixture, which was then kept at room temperature for 90 minutes. The absorbance of the samples was determined at a wavelength of 760 nm. Finally, the total phenolic content was calculated based on the standard curve as milligrams of gallic acid per gram of dry weight [22].

#### Measurement of total flavonoid content.

The total flavonoid content was measured using the aluminum chloride colorimetric method, with quercetin as the standard. A volume of 0.5 mL of the extract solution (prepared in the same manner as for the total phenol assay of leaves) was mixed with 150 µL of 95% methanol, 100 µL of 10% aluminum chloride, 100 µL of 1 M potassium acetate, and 280 µL of distilled water. After keeping the samples at room temperature for 30 minutes, the absorbance of the mixture was read at a wavelength of 415 nm. Quercetin was used to construct the standard curve, and the results were expressed as milligrams of quercetin per gram of dry leaf. By placing the absorbance value of the extract into the linear equation of the standard curve, the total flavonoid content in the leaf was calculated, and the data were ultimately expressed as the equivalent of milligrams of quercetin per gram of leaf [23].

### Evaluation of total yield

The harvest was conducted at the beginning of the flowering phase. For this purpose, an area equivalent to one square meter was harvested and weighed. After separating the leaves and stems, the samples were dried in an oven at 70°C for 48 hours. The total dry weight, as well as the dry weight of leaves and stems, were measured using a precision scale with an accuracy of 0.001 grams.

## Results

### Gene expression

The expression changes of genes involved in the TIAs pathway can be assessed through four different pathways. The indole, terpenoid, and alkaloid pathways are significant because they produce the final products of the pathway, while the phenolic and flavonoid compound production pathway is important as it competes with the indole pathway for the chorismate precursor [1–4]. Therefore, in the present study, the relative expression of some key genes in all four pathways was evaluated in the leaf tissue of the periwinkle plant (*Catharanthus roseus*).

**Fig 4:**
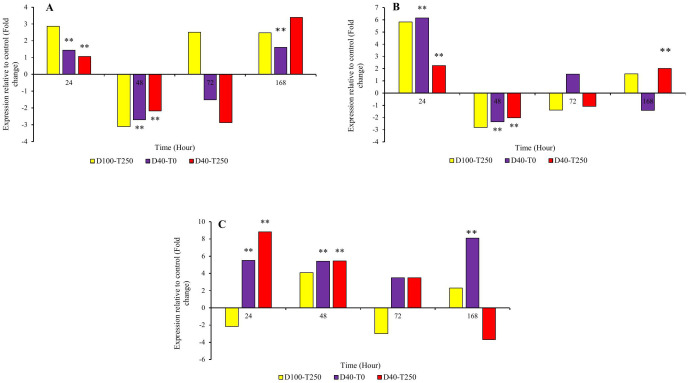
The graph of changes in the relative expression of A, B and C, respectively, *Cm*, *As* and *Tdc* gene over time (24, 48, 72 and 168 hours). D100.T250, D40.T0 and D40.T250, respectively: combined treatment of 250 mg/liter tryptophan and drought 100 crop capacity, combined treatment of zero mg/liter tryptophan and drought 40% crop capacity and combined treatment of 250 mg/liter tryptophan and drought 40% of agricultural capacity. *: significance at the 95% statistical confidence level and **: significance at the 99% statistical confidence level.

In general, the expression pattern of the chorismate mutase (*Cm*) gene, which is one of the primary genes in the pathway of phenolic and flavonoid compound production, showed an increase in relative expression in all three conditions: foliar application of 250 mg/L tryptophan, 40% drought stress, and the combined treatment of tryptophan and 40% drought stress, 24 hours after the application of the treatment and stress. Interestingly, a decrease in expression was observed at 48 and 72 hours after treatment, followed by an increase at 168 hours compared to the control. The only exception was the tryptophan treatment at 48 and 168 hours, which showed increased expression compared to the control (Fig 4-A). The highest relative expression was recorded in the combined tryptophan-drought treatment at 168 hours post-treatment (3.39), and in the tryptophan treatment 24 hours post-application with a relative expression value of 2.86.

In the indole alkaloid pathway, the anthranilate synthase (*As*) gene is located at the beginning, and the tryptamine decarboxylase (*Tdc*) gene is positioned at the end of the pathway. Notably, the relative expression changes of the *As* gene exhibited a pattern similar to the *Cm* gene, showing an increase in relative expression at the 24-hour time point, a decrease at the 48-hour and 72-hour time points, and another increase at the 168-hour time point across all three conditions. The only exceptions were observed at the 72-hour and 168-hour time points under drought stress (Fig 4-B). The highest relative expression levels under drought stress and tryptophan treatment at the 24-hour time point were recorded as 6.15 and 5.82, respectively. For the *Tdc* gene, relative expression changes showed a down- regulate at the 24-hour and 72-hour time points following tryptophan treatment and at the 168-hour time point under tryptophan-drought treatment compared to the control. Other treatment conditions showed up- regulated (Fig 4-C). The highest expression level for this gene was calculated as 8.82 under tryptophan-drought treatment at the 24-hour time point and 8.11 at the 168-hour time point following drought stress.

**Fig 5:**
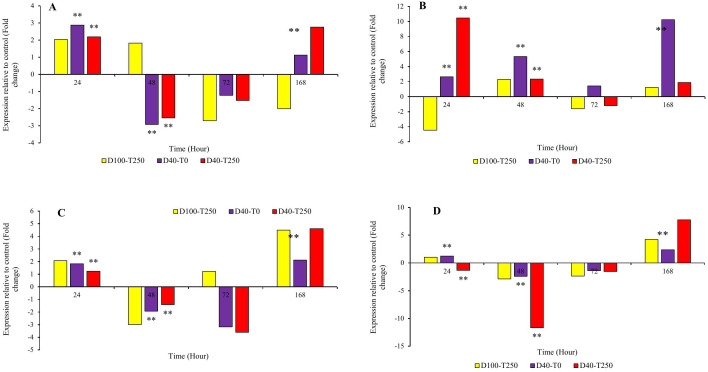
The graph of changes in the relative expression of A, B, C and D, respectively, *Sls*, *Str*, *Dat* and *Prx* gene over time (24, 48, 72 and 168 hours). D100.T250, D40.T0 and D40.T250, respectively: combined treatment of 250 mg/liter tryptophan and drought 100 crop capacity, combined treatment of zero mg/liter tryptophan and drought 40% crop capacity and combined treatment of 250 mg/liter tryptophan and drought 40% of agricultural capacity. *: significance at the 95% statistical confidence level and **: significance at the 99% statistical confidence level.

From the terpenoid pathway, changes in the expression of the secologanin synthase (*Sls*) gene, as a key gene in the mentioned pathway, were calculated. A 24-hour treatment period for all three conditions, a 48-hour treatment period after the application of tryptophan, and a 168-hour treatment period for drought stress and tryptophan-drought stress showed increased expression (Fig 5-A). In other treatment levels, a decrease in expression compared to the control treatment was observed. The highest relative expression values were calculated under drought stress at 24 hours (2.88) and under tryptophan-drought stress at 168 hours after treatment (2.76).

The alterations in the relative expression of the Strictosidine Synthase gene, as the first gene in the TIA alkaloid pathway, and the two genes Deacetylvindoline-4-O-acetyltransferase (*Dat*) and Peroxidase (*Prx*), as the final genes in the pathway, were evaluated. The analysis of *Str* gene expression changes in all time treatments and under all three conditions showed an increase in relative expression compared to the control treatment, except for the application of tryptophan at 24 and 72 hours and tryptophan-drought at 72 hours (Fig 5-B). Accordingly, the highest expression values were observed in the tryptophan-drought treatment at 24 hours post-treatment and drought stress at 168 hours post-stress application, with relative expression values of 10.45 and 10.24, respectively.

In a general overview, examining the trend of changes in the *Dat* gene was similar to the changes in most of the genes in the studied pathway. It showed up- regulate in all three conditions for the 24-hour treatment, and down- regulate for the 48-hour and 72-hour treatments, then again an increase in relative expression for the 168-hour treatment (Fig 5-C). The highest expression values, 4.5 and 4.62, corresponded to the treatments involving tryptophan and tryptophan-drought at 168 hours after treatment application, respectively. The lowest relative expression values were associated with the tryptophan-drought treatment at 72 hours, drought stress at 72 hours, and the tryptophan treatment at 48 hours, with expression values of −3.61, −3.17, and −2.99, respectively.

In the evaluation of the relative expression of the *Prx* gene, the highest increase in expression was observed at the 168-hour treatment time for all three conditions compared to the control treatment (Fig 5-D). The tryptophan-drought treatment and tryptophan treatment showed the highest relative expression levels, with expression values of 7.76 and 4.23 respectively, 168 hours after treatment. Meanwhile, the tryptophan-drought treatment exhibited the lowest relative expression (−11.65) 48 hours after treatment.

### Evaluation of vinblastine and vincristine alkaloid content

**Fig 6:**
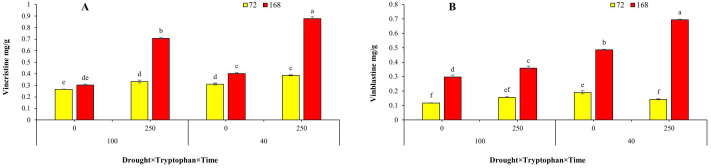
Comparison of the response of drought stress treatments (100 and 40% of field capacity) to different amino acids tryptophan (0 and 250 mg/L) at different times (72 and 168 hours), based on vincristine (A) and vinblastine (B) traits. Columns with the same letters are not significantly different based on Duncan’s range test at the 5% error probability level.

The results of the analysis of variance indicated that the effects of drought stress and the foliar application of the plant C. *roseus* with the amino acid tryptophan at various sampling times significantly influenced the concentrations of vincristine at the 5% significance level and vinblastine at the 1% significance level (Table 2). Both drought stress and the foliar application of tryptophan resulted in increased levels of vincristine and vinblastine in the C. *roseus* plant. Mean comparison analyses revealed that the highest concentrations of vincristine and vinblastine, measured at 0.87 and 0.69 mg/g of dry leaf weight, respectively, were achieved at a concentration of 250 mg per liter under severe drought stress (40% field capacity) after 168 hours. These concentrations correspond to increases of 230% for vincristine and 488% for vinblastine when compared to the control treatment. In contrast, the lowest concentrations of vincristine and vinblastine, recorded at 0.26 and 0.11 mg/g of dry leaf weight, respectively, were observed in the control treatment, which involved a zero tryptophan concentration and no drought stress (100% field capacity) at the 72-hour sampling point (Fig 6A and B).

**Table 2:** Results of the analysis of variance (ANOVA) regarding the effects of drought stress treatment and the amino acid tryptophan at various time intervals on the alkaloids vincristine and vinblastine in the periwinkle plant.

S.O.V	D.F	Mean squares	
		Vincristine	Vinblastine
Drought	1	0.05^**^	0.12^**^
Tryptophan	1	0.39^**^	0.02^**^
Time	1	0.37^**^	0.56^**^
Drought × Tryptophan	1	0.002^**^	0.001^*^
Drought × Time	1	0.011^**^	0.080^**^
Tryptophan × time	1	0.20^**^	0.029^**^
Drought × Tryptophan × Time	1	0.001^*^	0.021^**^
Error	16	0.00021	0.0002
C.V (%)	–	3.3	4.69

ns, **, and * indicate non-significant, and significant at the 1% and 5% error probability levels, respectively.

### Evaluation of non-enzymatic antioxidant levels

The results of the analysis of variance for greenhouse data showed that the interaction effect of drought stress with the amino acid tryptophan over time on the traits of phenol and flavonoid was significant at a 5% error probability level. However, the interaction effect of drought and tryptophan on the phenol trait was not significant (Table 3).

**Table 3:** Analysis of variance results for the effect of drought stress treatment and tryptophan amino acid on the physiological indices of the periwinkle plant.

S.O.V	D.F	Mean squares	
		Phenol	Flavonoid
Drought	1	103.7^**^	4.4^**^
Tryptophan	1	50.4^**^	2.36^**^
Time	3	7.81^**^	5.76^**^
Drought × Tryptophan	1	1.08^ns^	2.47^**^
Drought × Time	3	0.98^*^	4.12^**^
Tryptophan × time	3	.86^**^	0.57^ns^
Drought × Tryptophan × Time	3	3.22^*^	0.87^*^
Error	30	0.31	0.28
C.V (%)	–	6.01	22.9

ns, **, and * indicate non-significant, and significant at the 1% and 5% error probability levels, respectively.

**Fig 7:**
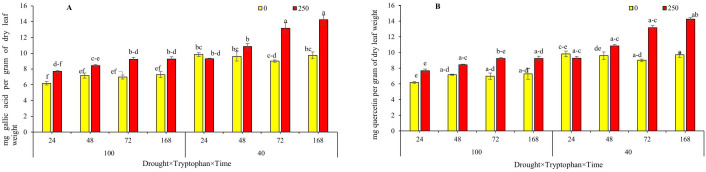
Comparison of the mean response of drought stress treatments (100 and 40% of field capacity) with different concentrations of the amino acid tryptophan (0 and 250 mg/L) at various times (24, 48, 72 and 168 hours) in terms of phenol (A) and flavonoid (B) traits. Columns with similar letters are not significantly different at the 5% error probability level according to Duncan’s test.

The results of the analysis of variance table showed that the effect of drought stress and foliar application of vinca plant with the amino acid tryptophan at different sampling times on the concentration of phenol and flavonoid was significant at the 5% probability level (Table 3). Drought stress and foliar application of tryptophan amino acid increased the phenol and flavonoid content in the vinca plant. The mean comparison results indicated that the highest phenol content (14.25 mg gallic acid per gram of dry leaf weight) was obtained from a concentration of 250 mg/L at 168 hours, and the highest flavonoid content (3.65 mg quercetin per gram of dry leaf weight) was achieved at zero concentration (control) under severe drought stress (40% field capacity) at 168 hours. Additionally, the lowest phenol content (6.19 mg gallic acid per gram of dry leaf weight) and flavonoid content (0.34 mg quercetin per gram of dry leaf weight) were observed at zero concentration without drought stress (100% field capacity) within 24 hours (Fig 7A, B).

### Dry weight of the plant

**Fig 8:**
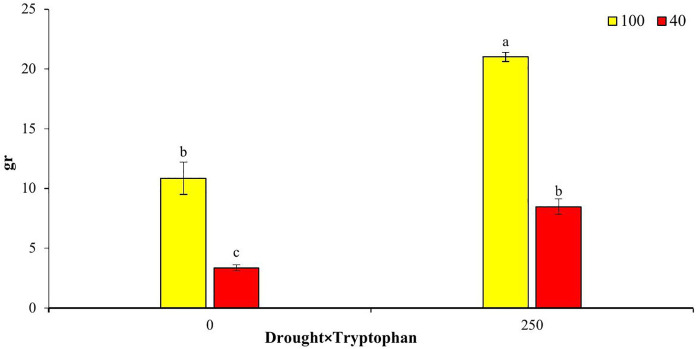
Comparative analysis of drought stress treatment response with the amino acid tryptophan concerning total plant dry weight trait.

The results of the mean comparisons indicated that increasing the concentration of tryptophan and soil moisture had a positive and significant effect on the dry weight of the medicinal plant bush. As the concentration of the tryptophan amino acid increased, the dry weight of the bush also increased. The highest dry weight recorded was 21 grams, achieved with the application of 250 mg/L of tryptophan at a moisture level equivalent to 100% field capacity, representing a 93.3% increase compared to the control group. Conversely, a reduction in tryptophan concentration to zero (control) combined with an increase in drought stress to 40% field capacity resulted in a decrease in the dry weight of the bush, with the lowest average dry weight recorded at 3.7 grams, which corresponded to a 69.6% decrease compared to the control (Fig 8).

## Discussion

Drought stress and water scarcity constitute significant challenges for global agriculture. Under conditions of drought stress, plants enhance their tolerance by accumulating organic osmolytes, including polyols, proline, and betaines [11,24]. The application of specific elicitors can bolster plant tolerance to stress, with the amino acid tryptophan being a notable example. Research indicates that tryptophan serves as a precursor to auxin hormones and exerts a more substantial influence on growth and performance compared to the external application of auxins [11,25]. Auxins, recognized as phytohormones, also function as osmotic protectants, playing a critical role in alleviating the adverse effects of drought stress on plant growth [26]. In addition to its classification as an osmolyte, tryptophan regulates ion transport, detoxifies heavy metals, mitigates their detrimental effects, and modulates stomatal opening auxins [11,25]. In our study, the foliar application of tryptophan resulted in the highest dry weight of both the aerial parts and roots of the plant. Furthermore, the application of this amino acid mitigated the impacts of drought stress, leading to an increase in dry weight under drought conditions. These findings are consistent with numerous studies in the field of drought stress and the application of growth stimulants. For instance, the foliar application of L-arginine under drought stress has been reported to significantly enhance traits such as water consumption, water use efficiency, and plant yield, with the optimal yield achieved at 100% field capacity and 3 (mM) L-arginine [27]. Besides to the beneficial effects of L-arginine under drought stress, the application of tryptophan also increased relative water content, osmotic potential, and chlorophyll levels in plants subjected to drought stress [11]. The research presented suggests that the application of tryptophan activates the synthesis of carbohydrates and hormones, such as auxin, which promotes root growth and nutrient uptake, ultimately leading to enhanced growth indices in plants under abiotic stresses, including drought stress [14,28].

Moreover, the influence of tryptophan on the expression patterns of genes involved in three pathways indole (*As* and *Tdc* genes), terpenoid (*Sls*), and alkaloid (*Str*, *Dat*, and *Prx* genes) associated with the TIAs pathway and the competing pathway (which produces phenolic and flavonoid compounds *Cm* gene) was evaluated. Chorismate mutase (CM) competes with anthranilate synthase (*AS*), which is encoded by the first gene in the indole pathway, for the chorismate substrate. The outcome of this competition determines whether chorismate is directed toward the indole and TIAs pathway or towards the biosynthesis of flavonoids and phenylpropanoids. If chorismate, due to the activity of chorismate mutase, is funneled into the flavonoid and phenylpropanoid synthesis pathway, the reaction pathway shifts from the production of the desired indole terpenoid alkaloids to the synthesis of phenylalanine, tyrosine, and ultimately, flavonoid and phenylpropanoid compounds. Conversely, if the enzyme anthranilate synthase is active, the activation of the indole pathway (TIAs) occurs, which will, in turn, influence the activity of downstream genes and the synthesis of alkaloids within that pathway [4,17]. The investigation of alterations in the relative expression patterns of both *Cm* and *As* genes under three distinct conditions (treatment with 250 mg per liter of tryptophan, 40% drought stress, and the combined treatment of tryptophan and 40% drought stress) demonstrated an initial increase in expression at the 24-hour treatment mark, followed by a declining trend at 48 and 72 hours. Notably, at 168 hours, an increase in expression relative to the control was observed. Additionally, the assessment of phenol levels resulting from tryptophan application indicated a continuous upward trend, which aligns with the observed changes in the relative expression of the *Cm* gene. In general, plants subjected to stress synthesize compounds that contribute to enhanced tolerance or adaptation to adverse conditions. Consequently, the production of non-enzymatic phenolic-derived compounds constitutes one of the strategies employed by plants in response to stressors such as drought. Despite the generation of both enzymatic and non-enzymatic compounds as defensive mechanisms, the application of specific elicitors bolsters the plant’s defense system and accelerates its response [24,25,29,30]. Furthermore, phenolic compounds and flavonoids serve as crucial non-enzymatic antioxidants that facilitate the neutralization of reactive oxygen species (ROS) and inhibit lipid peroxidation, thereby playing a significant role in alleviating the impacts of stress [31]. The drought stress-induced increase in phenylalanine ammonia-lyase enzyme activity and the consequent production of phenolic and flavonoid compounds in various plants, including primrose [32], dill [33], black cumin [34,35], marigold [36], rosemary [37], and sage [38], has been substantiated, underscoring the modulation of detrimental effects resulting from stress. The application of phenylalanine and tryptophan amino acids as osmotic regulators and primary signaling molecules under stress conditions has been noted to enhance the activity of phenylalanine ammonia-lyase. This behavior, through the upregulation of the relative expression of its synthesizing gene, results in the biosynthesis and accumulation of phenolic compounds, thereby contributing to the mitigation of stress’s adverse effects in plants. Our findings suggest that tryptophan application not only exerts a beneficial influence on overall plant performance but also positively affects secondary metabolites such as phenols and flavonoids, facilitating the modulation of stress effects. In the production of medicinal alkaloids in the plant Periwinkle, alterations in gene expression within the terpenoid pathway, alongside the indole pathway and its competing pathways, are also significant. Secologanin synthase (SLS), the terminal enzyme in the terpenoid pathway of terpenoid indole alkaloids (TIAs), is responsible for converting loganin to secologanin. The activity of the STR enzyme enables secologanin, produced through SLS enzyme activity, along with tryptamine generated from TDC enzyme activity in the indole pathway of TIAs, to enter the alkaloid pathway of TIAs [6,16]. The assessment of relative expression changes of the *Sls* gene, akin to other genes studied in this research, demonstrated an increase in expression at the 24-hour treatment mark across all three conditions. However, a decrease in expression was observed at the 48 and 72-hour intervals, while an increase was noted at 168 hours, except in the case of tryptophan spraying at 48 and 168 hours, which resulted in an increase and decrease, respectively. The variations in this gene further indicated the impact of tryptophan application in both drought stress conditions and non-stress conditions. In C. *roseus* cell culture, Cyclodextrin treatments and the synergistic effects of methyl jasmonate and Cyclodextrin resulted in a significant increase in the expression of the *Sls* gene compared to the control, with peak transcription levels observed on the first and fifth days. As anticipated, changes in the relative expression of genes within each signaling pathway, including the TIAs pathway, can influence the final product levels of that pathway. This study demonstrated that the elevation in expression levels of pathway genes correlated with increased quantities of the alkaloids ajmalicine and catharanthine [16]. The transcription levels of the *Tdc* gene in the seedlings of the butterfly plant exhibited an increase in response to methyl jasmonate (MeJA) treatment. This increase demonstrated a progressive upward trend over time, with the maximum transcription levels observed in treatments with concentrations of 0.2 and 2 mM at 48 hours post-treatment application [15]. Considering the positioning of genes within the biosynthetic pathway of tertiary indole alkaloids (TIAs), alterations in the transcription levels of any upstream genes may consequently affect the transcription levels of downstream genes. Existing literature indicates that an increase in the transcription level of the *Tdc* gene, which is involved in the indole TIAs pathway, or an elevation in the transcription level of the *Sls* gene, associated with the terpenoid TIAs pathway, can lead to an increase in the synthesis of alkaloids within the downstream pathway [4,8,16,17]. A comprehensive overview reveals that, aside from minor variations in expression across certain time treatments, the expression of all three genes implicated in the alkaloid pathway (*Str*, *Dat*, and *Prx*) exhibited significant up-regulated at 24 and 168 hours when compared to the control treatment. The gene encoding strictosidine synthase (STR), the initial gene in the TIAs alkaloid pathway, along with the genes *Dat* and *Prx*, acts as terminal genes in the pathway that facilitate the conversion of Deacetylvindoline to vindoline, a precursor for the synthesis of vinblastine and vincristine [4,6]. Reports suggest that the up-regulation of these genes correlates with the accumulation of vindoline and the commercially significant alkaloids vincristine and vinblastine [17,39–41]. The overexpression of the *Dat* gene, as demonstrated in the study conducted by Wang et al. (2012), along with quantitative reverse transcription polymerase chain reaction (qRT-PCR) analyses, revealed increased expression levels of the *Dat* gene across all transgenic plants. This finding aligns with the observed accumulation of vindoline in the studied plants, thereby confirming the positive impact of heightened transcription levels of this gene on vindoline production [41]. Investigations into the effects of methyl jasmonate and putrescine on the expression of the *Dat* gene, as well as the levels of the alkaloids vincristine and vinblastine, indicated that these treatments resulted in an up-regulation of the *Dat* gene and a corresponding increase in the aforementioned alkaloids [39]. Furthermore, the foliar application of salicylic acid at concentrations of 5–10 and 4–10 molar led to enhanced relative expression of the genes implicated in the TIA pathway (*Cm*, *As*, *Tdc*, *G10h*, *Sls*, *Str*, *D4h*, and *Dat*) [42] and ultimately increased the quantities of the pathway’s two end products (vincristine and vinblastine) in the periwinkle plant [17]. Based on research conducted on the TIA signaling pathway in the periwinkle plant, it is anticipated that shifts in the expression patterns of the pathway genes will also influence the levels of the alkaloids vincristine and vinblastine, consistent with findings from numerous studies related to the TIA pathway, irrespective of the elicitor type or stress applied [4,8,17,39,42–44]. The results obtained from high-performance thin-layer chromatography (HPLC) in this study indicate that the treatments applied not only activate gene expression but also influence the levels of the alkaloids vincristine and vinblastine. The application of amino acids, particularly tryptophan, contributes to alleviating stress conditions and demonstrates an increase in these compounds. Drought stress, in conjunction with the application of tryptophan, significantly enhances the production of vincristine and vinblastine in the periwinkle plant. Under severe drought stress conditions (40% field capacity) combined with 250 mg/L of tryptophan, the levels of vincristine and vinblastine in dried leaves increased compared to the control group. These findings suggest that environmental stresses, particularly drought, can stimulate the synthesis of secondary metabolites in medicinal plants. Conversely, the lowest levels of vincristine and vinblastine were observed in the control treatment with zero tryptophan under non-stress conditions (100% field capacity) within 72 hours. This clearly demonstrates that the absence of tryptophan under non-stress conditions minimizes the production of these metabolites, thereby rendering the plants more vulnerable to environmental conditions. Indeed, the low levels of these compounds in the treatment may be attributed to factors influencing the biochemical pathways associated with the production of secondary metabolites. This study aligns with the findings of previous research on the periwinkle plant [45–47].

## Conclusions

Based on the results obtained from the present study, the application of tryptophan as a foliar treatment on the plant *Catharanthus roseus* not only alleviates the adverse effects of drought stress but also exerts a positive influence on the plant’s dry weight performance, the expression of genes within the terpenoid indole alkaloids (TIAs) biosynthetic pathway, and the production of valuable alkaloids. This treatment has the potential to enhance both agricultural yield and the quantities of the alkaloids vincristine and vinblastine. Therefore, tryptophan foliar application can be regarded as a recommended intervention for improving the commercial production of alkaloids in Periwinkle and as a viable strategy for addressing agricultural challenges associated with water scarcity.
