# Supplementary material for: Evaluation of the expression pattern of TIAs pathway genes in response to tryptophan amino acid treatment and drought stress in Catharanthus roseus
Source: PLoS One. 2025 Oct 9;20(10):e0333313. doi: 10.1371/journal.pone.0333313 (PMC12510576; doi:10.1371/journal.pone.0333313)
Supplement: Attachment — Submitted filename: Response to Reviewers- PONE-D-25-31312.docx [file pone.0333313.s004.docx]

Dr. Ajit Prakash
Academic Editor
PLOS ONE

September 2025

Dear Dr. Ajit Prakash

We sincerely thank you for considering our article for publication in the journal "PLOS ONE." Based on your valuable recommendations and with respect to the constructive comments of the reviewers, we have already revised our manuscript accordingly. Additionally, all the reviewers' comments mentioned in this letter have been taken into account. The article has been re-checked for grammar and spelling. Moreover, all data and figures are included in the manuscript and the supporting information files. Regarding Figure 2, the esteemed author Dr. Soltani designed it, and the design steps are also uploaded in the PowerPoint file along with the other files. Finally, the last reference in the reference list is highlighted in yellow according to the Vancouver style.

Thank you for your time and assistance.

Yours sincerely,

Farshid Yousefi

Corresponding author of the article

Agricultural Sciences and Natural Resources University of Khuzestan

**Reviewer 1:**

**We appreciate the keen observation of the respected reviewer. Your suggested changes are highlighted in yellow in the manuscript.**

**Minor points:**

**Point 1:**

Page 5 – Line 108 – Figure 2: The phrase “poplin related to the study stages” in the figure title appears unclear and could benefit from rewording for improved clarity and relevance to the experimental context.

**Response:**

We are grateful to the respected reviewer to pointing this out to us. We corrected it in text.

**Point 2:**

Page 7 – Line 149 – Table 1: The annealing temperature is labeled as “AM,” which may be a typographical error. It would be more appropriate to use the conventional abbreviation Ta.

**Response:**

We appreciate the keen observation of the respected reviewer. Our false claim was removed.

**Point 3:**

Pages 10 to 13 – Figures 4 and 5: In the bar graphs, the Y-axis is labeled “Experation relative to control.” This seems to be a typographical error and should be corrected to “Expression relative to control.”

**Response:**

Thank you to the reviewer for pointing out this important point. Done.

**Reviewer 2:**

Dr. Sounak Ghosh Roy

We are grateful for your valuable comments and the time you took to review our manuscript.
